# Revisiting the link between domain satisfaction and life satisfaction during the COVID-19 pandemic: Job-related moderators in triadic analysis in dual-earner parents with adolescent children

**DOI:** 10.3389/fpubh.2023.1108336

**Published:** 2023-02-06

**Authors:** Berta Schnettler, Andrés Concha-Salgado, Ligia Orellana, Mahia Saracostti, Edgardo Miranda-Zapata, Héctor Poblete, Germán Lobos, Cristian Adasme-Berríos, María Lapo, Katherine Beroíza, Leonor Riquelme

**Affiliations:** ^1^Facultad de Ciencias Agropecuarias y Medioambiente, Universidad de La Frontera, Temuco, Chile; ^2^Scientific and Technological Bioresource Nucleus (BIOREN-UFRO), Universidad de La Frontera, Temuco, Chile; ^3^Centro de Excelencia en Psicología Económica y del Consumo, Universidad de La Frontera, Temuco, Chile; ^4^Universidad Católica de Santiago de Guayaquil, Guayaquil, Ecuador; ^5^Departamento de Psicología, Universidad de la Frontera, Temuco, Chile; ^6^Escuela de Trabajo Social, Universidad de Valparaíso, Valparaíso, Chile; ^7^Facultad de Educación, Centro de Investigación Escolar y Desarrollo (CIED-UCT), Universidad Católica de Temuco, Temuco, Chile; ^8^Universidad Autónoma de Chile, Temuco, Chile; ^9^Facultad de Economía y Negocios, Universidad de Talca, Talca, Chile; ^10^Departamento de Economía y Administración, Universidad Católica del Maule, Talca, Chile; ^11^Doctorado en Ciencias Sociales, Universidad de La Frontera, Temuco, Chile

**Keywords:** life satisfaction, job, family, food, spillover, crossover, moderating role

## Abstract

**Introduction:**

Research has evaluated the impact of COVID-19 pandemic and lockdowns on individuals' life satisfaction, but wellbeing interrelations between family members in this context have been less explored. This study examined the spillover and crossover effects of one parent's job satisfaction (JS), satisfaction with family life (SWFaL) and satisfaction with food-related life (SWFoL) on their own, their partner's, and their adolescent children's life satisfaction (LS), and the influence of adolescents' SWFaL and SWFoL on their own and their parents' LS, in dual-earner families with adolescents. The moderating role of job-related variables of both parents were also explored.

**Methods:**

Questionnaires were administered to 860 dual-earner parents with adolescents in two cities in Chile during 2020. Mothers and fathers answered the Overall Job Satisfaction Scale and the three family members answered the Satisfaction with Life Scale, the Satisfaction with Family Life Scale and the Satisfaction with Food-related Life Scale.

**Results:**

Using the Actor-Partner Interdependence Model and structural equation modeling, we found that fathers' LS was positively associated with their own JS, SWFaL and SWFoL (spillover), and negatively with adolescents' SWFoL (crossover). Likewise, mothers' LS was positively associated with their own JS, SWFaL and SWFoL (spillover), with fathers' and adolescents' SWFaL, and negatively with adolescents' SWFoL. Adolescents' LS was positively associated with their own SWFaL and SWFoL (spillover), and with their fathers' JS, and negatively with their fathers' SWFoL. JS showed gendered patterns in spillover and crossover associations. Parents' type of employment, mothers' working hours and city of residence moderated some spillover and crossover associations for father-mother and parent-adolescent dyads.

**Discussion:**

These findings suggest that, for dual-earner parents with adolescents, improving individuals' LS requires interventions that should be carried out not individually, but at a family level.

## 1. Introduction

The COVID-19 pandemic and its lockdown measures to reduce the risk of infection has changed daily life for individuals and their families (1–3). Family responsibilities have multiplied for parents working from home, as school and childcare facilities closed during lockdown (4), and parents blurred the boundaries between their work and non-work domains (5). Moreover, the impact of the pandemic on children and adolescents has been mostly addressed through mental health constructs such as depression or anxiety, while life satisfaction and its determinants have been less studied during this period (6, 7). This study focuses on families of dual-earner parents with adolescents because these parents have faced additional challenges in their work-family interface (8) while adolescents continue to benefit from parental support as they are also aware of how work affects their parents' lives (9, 10).

The bottom-up approach to life satisfaction proposes that overall life satisfaction depends on a person's level of satisfaction in different life domains (11). Life satisfaction, or the cognitive component of subjective wellbeing (12), has been found to be unaffected by the COVID-19 pandemic in some samples (2, 13), and negatively affected in others [e.g., (1, 5, 7)], especially women and parents (14). Researchers also show that the pandemic has decreased domain satisfaction, most notably family life satisfaction, or the conscious cognitive judgment of one's family life (15), and job satisfaction, the extent to which workers like their job (16). This decrease has been mainly reported in mothers (5). A third domain of interest during the pandemic is that of food. Satisfaction with food-related life (17) is a person's overall cognitive assessment of their food and eating habits, including, but not limited to, diet quality. In this latter domain, findings obtained during the pandemic vary, as some show an increased diet quality (8, 18), while others show a decreased diet quality (19), or no significant changes in diet quality (20).

Most available studies assessing the influence of domain satisfaction on life satisfaction have been conducted at an individual level, overlooking potential interrelations between family members. Family systems theory (21) focuses on this interdependence, proposing that family members are involved in reciprocal relationships with one another. In line with this theory, the “spillover-crossover” model [SCM, (22)] posits that experiences in one life domain affect another domain or overall life satisfaction, at an intra-individual and inter-individual level. “Spillover” refers to the intraindividual transmission of experiences, while “crossover” refers to a dyadic, inter-individual transfer of experiences (22). Studies have reported unidirectional or asymmetric crossover effects, from one partner to the other [e.g., (23, 24)], but also bidirectional or symmetric effects, from one partner to the other and vice versa [e.g., (25–27)]. Studies on parent-child dyads, have also reported crossover from only one parent to their children [e.g., (10, 18)] and from both parents to their children [e.g., (28, 29)]. Research on crossover from children to their parents are sparser (30, 31).

On this basis, this study focused on the contribution of satisfaction in the job, family, and food domains on the overall life satisfaction during the first year of the pandemic. The unit of analysis is the triad of family members composed of dual-earner parents and one of their adolescent children. The relationships between these domains have gained even more relevance during the COVID-19 pandemic, as this context has highlighted the roles of the nuclear family (32); it has blurred the boundaries between work and domestic responsibilities (33); and it has increased family coexistence, for instance, as families get together more frequently for meals (18, 20). This study builds on the bottom-up life satisfaction approach, family systems theory, and the SCM. The analysis is supported by the Actor-Partner Interdependence Model [APIM, (34)]. The APIM tests actor effects or spillover, and partner effects or crossover; actor effects are outcomes predicted by individuals' own characteristics, and partner effects are outcomes from one member of a dyad predicted by the characteristics of the other member (34). The APIM also allowed to further explore the role of gender, as studies have found different gender patterns for job, family, and food-related life satisfaction in their association with life satisfaction [e.g., (24, 35, 36)]; and it allowed to fill in a knowledge gap regarding potential job-related moderators in the relationships between life domains and life satisfaction (18).

In keeping with the bottom-up approach, researchers have thus identified three relevant domains that contribute to life satisfaction. First, the job domain is one of the most relevant aspects of an adult's life as it takes up a large share of their time (37, 38). The degree of importance of the job domain as a contributor to life satisfaction has mixed evidence (37–40), but several studies support a positive spillover between job satisfaction and life satisfaction in workers in different countries [e.g., (41–43)]. Furthermore, this contribution of job satisfaction to life satisfaction has been reported before (39, 40) and during the pandemic (5, 44). There is also evidence of crossover effects between job-related variables and life satisfaction among different-gender dual-earner couples with adolescents (24, 25, 36, 45). Some of these studies have found asymmetrical effects (i.e., from one partner to the other but not vice versa), hypothesizing that gender dynamics may explain these differential effects. Studies also report crossover effects in parent-child dyads, such that mothers' positive experiences at the workplace positively influence their children's life satisfaction and wellbeing (10, 46), and that both parents' work-life balance positively influences their adolescents' life satisfaction (47, 48).

A second potential contributor to life satisfaction is the family domain (39, 47, 49). Studies show that having good relationships within the family, family support and a positive family functioning result in higher family and life satisfaction in adults and adolescents (24, 32, 47, 48). Research also supports a positive spillover from satisfaction with family life (SWFaL) and overall life satisfaction, in adults (40, 44, 50) and adolescents (2, 18, 32, 47, 49, 51), before and during the pandemic. Crossover effects between family members' SWFaL and life satisfaction have been less researched. However, family-related variables have shown crossover effects in father-mother dyads (18, 35, 36), and in mother-adolescent dyads (10). These crossover effects may have become more pronounced during the pandemic, hindering or benefitting relationships between family members (3).

A third potential contributor to life satisfaction is the food domain, which has gained attention in recent years. The role of satisfaction with food-related life (SWFoL) has been less studied, but pre-pandemic evidence suggests that it is associated with life satisfaction in adult and adolescent samples (18, 47, 49, 52). Diet quality, a variable linked to this domain, has been positively associated with satisfaction with food-related life [SWFoL, (18, 28, 53)]. Crossover effects have been found between SWFoL and life satisfaction from fathers to mothers (54), and from adolescents to mothers (31).

Overall, this evidence suggests that a parent's life satisfaction is influenced by their own satisfaction in different life domains, and by those of the other parent and their children. Likewise, children's life satisfaction may not only be influenced by their own satisfaction in different life domains, but also by those of their parents.

Spillover and crossover effects between domain satisfaction and life satisfaction may differ among men and women. Both pre-pandemic studies (55), and studies carried out during the pandemic show that men report higher levels of life satisfaction than women (13, 56). This difference may be due to how men and women are socialized to relate to determinants of life satisfaction [e.g., (57)]. In this sense, gender role theory states that roles in society differ by the socialization practices according to gender, such as that work roles are fundamental to men's identities, and family roles are more significant to women's identities (58). This is a predominant viewpoint in the cultural context of this study, in which men are considered the main providers for the family (27), while women are identified as primary caregivers (9, 10, 59). Studies on crossover effects by gender show more frequently unidirectional crossover from husbands to wives (23–25, 35, 36, 60), possibly in keeping with women's traditional socialization to be more sensitive to conditions affecting their male partner (23, 31, 60–62).

However, other studies show that the main life satisfaction determinants are similar for men and women (36, 63). Moreover, while spillover effects tend to be stronger than crossover effects among dyads (60, 62, 64), gender differences in these effects appear to depend on the variables analyzed (25, 36, 45, 65). Studies with Chilean dual-earner couples (25, 36) have shown that the second contributor to life satisfaction is SWFoL for women, and job satisfaction for men, but the primary contribution for both groups is SWFaL. This result may relate to the high relevance of family life in Latin American cultures (66).

Lastly, research has shown that job-related variables are related to life, job, family life and food-related life satisfaction. Self-employed workers have reported higher levels of life (67), job (68, 69), family life (70) and food-related life satisfaction (71) than those who are formal employees. Moreover, Loewe et al. (38) have found a positive association between job and life satisfaction, and this relationship is stronger for self-employed workers than for employees. Part-time employees have also reported greater levels of job (72), and food-related life satisfaction (61) than those working full-time. In this line, more working hours in mothers have been linked to lower SWFoL in their adolescent children (71), and self-employed mothers and their adolescents children have reported better diet quality than employed mothers and their adolescents (28). During the COVID-19 pandemic, changes in adults' life satisfaction has been also associated with the city and region of residence, that is, the likelihood of experiencing decreased life satisfaction is higher for those living in a big city compared to those living in small cities or in rural areas (13, 73). However, the moderating role of parent's job-related variables and place of residence on domain and life satisfaction for the individual and their family members remain scarcely explored. To the best of the author's knowledge, there are no available studies testing the moderating role of both parents' job-related variables and place of residence on spillover and crossover associations for domain and life satisfaction in individuals', their partner's, and their adolescent's life satisfaction.

Against this background, the first aim of this study was to explore the spillover and crossover effects from one parent's job satisfaction, satisfaction with family life and satisfaction with food-related life on their own, the other parent's, and their adolescent children's life satisfaction, and the effects of adolescents' satisfaction with family life and with food-related life on their own and on their parents' life satisfaction. A second aim was to test differences in spillover and crossover effects according to the parent's gender. Lastly, this study explored the moderating role of job-related variables and city of residence in these relations.

The following hypotheses were posed:

H1: The father's life satisfaction is positively associated with his own (a) job satisfaction, (b) SWFaL, and (c) SWFoL (spillover effect).
H2: The mother's life satisfaction is positively associated with her own (a) job satisfaction, (b) SWFaL, and (c) SWFoL (spillover effect).
H3: The adolescent child's life satisfaction is positively associated with their own (a) SWFaL and (b) SWFoL (spillover effect).
H4: The father's life satisfaction is positively associated with the mother's (a) job satisfaction, (b) SWFaL, (c) SWFoL, and with the adolescent child's (d) SWFaL and (e) SWFoL (crossover effect).
H5: The mother's life satisfaction is positively associated with the father's (a) job satisfaction, (b) SWFaL and (c) SWFoL, and with the adolescent child's (d) SWFaL and (e) SWFoL (crossover effects).
H6: The adolescent child's life satisfaction is positively associated with the father's (a) job satisfaction, (b) SWFaL and (c) SWFoL, and with the mother's (d) job satisfaction, (e) SWFaL and (f) SWFoL (crossover effects).
Lastly, we proposed H7: The spillover relationship between job satisfaction and life satisfaction (a) for fathers is significantly higher than the crossover association between mothers' job satisfaction and the father's life satisfaction and (b) for mothers this spillover does not differ from the crossover association between fathers' job satisfaction and the mother's life satisfaction.
H8: The spillover relationship between each parent's SWFaL and life satisfaction is significantly higher than the crossover association between one parent's SWFaL and the other parent's life satisfaction for (a) fathers and (b) mothers.
H9: The spillover relationship between SWFoL and life satisfaction (a) for mothers is significantly higher than the crossover association between fathers' SWFoL and the mother's life satisfaction and (b) for fathers this spillover does not differ from the crossover association between mothers' SWFoL and the father's life satisfaction.
H10: The crossover effect of father's job satisfaction on the adolescent's life satisfaction is significantly higher than the mother's crossover effect (crossover effects by gender).
H11: The crossover effects of the mother's (a) SWFaL and (b) SWFoL on the adolescent's life satisfaction are significantly higher than the father's crossover effects (crossover effects by gender).

We also posed one research question:

RQ1. Do both parents' job-related variables (i.e., type of employment and working hours) and the city of residence moderate the spillover and crossover associations between each parent's job satisfaction, SWFaL, and SWFoL on their own, the other parent's, and the adolescent's life satisfaction?

## 2. Methods

### 2.1. Sample

We recruited a non-probability sample of 860 dual-earner families composed by mothers and fathers (married or cohabiting) with at least one child aged between 10 and 15 years, in the cities of Temuco and Rancagua. Families were recruited as part of a larger study on domain and life satisfaction in Chilean dual-earner families (47). For each city, sample size was determined considering 10 participants for each item of each scale used in this research project (74). Table 1 shows the sociodemographic characteristics for the 860 triads (mother-father-adolescent) and the average SWFaL, SWFoL, and job and life satisfaction scores. The mean age for mothers was 39.0, for fathers it was 42.2 and for adolescents 13.1 years (50.8% female). Families were composed of four members and two children living in the household on average, and most belonged to a middle SES. Regarding differences between cities, Temuco had a greater proportion of families belonging to the low SES and Rancagua a greater presence of families belonging to the middle SES (*p* < 0.001).

**Table 1:** Sample characteristics of participant families (*n* = 860).

**Characteristic**	**Temuco**	**Rancagua**	**Total sample**	***P*-value**
	**(*n* =430)**	**(*n* =430)**		
Age [Mean (*SD*)]^a^				
Father	42.0 (8.6)	42.3 (7.8)	42.2 (8.2)	0.607
Mother	38.6 (7.3)	39.4 (6.6)	39.0 (6.9)	0.091
Adolescent	13.1 (1.8)	13.2 (2.0)	13.1 (1.9)	0.723
Adolescent's gender^b^				
Male	52.1	46.3	49.2	0.088
Female	47.9	53.7	50.8	
Number of family members [Mean (*SD*)]^a^	4.3 (1.1)	4.3 (1.0)	4.3 (1.0)	0.921
Number of children living in the household [Mean (*SD*)]^a^	2.2 (0.9)	2.2 (0.9)	2.2 (0.9)	0.525
Socioeconomic status (%)^b^				
High	1.6	3.7	2.7	< 0.001
Middle	74.9	83.0	79.0	
Low	23.5	13.3	18.4	
Number of days/week couples ate together [Mean (*SD*)]^a^				
Breakfast	4.2 (2.7)	3.5 (2.7)	3.8 (2.7)	< 0.001
Lunch	5.2 (2.4)	4.9 (2.4)	5.1 (2.4)	0.052
Supper	6.0 (2.1)	6.0 (1.9)	6.0 (2.0)	0.919
Satisfaction with life (SWLS) [Mean (*SD*)]^a^				
Father	24.7 (4.2)	24.0 (4.9)	24.4 (4.5)	0.008
Mother	23.9 (4.5)	23.7 (4.6)	23.8 (4.6)	0.580
Adolescent	24.7 (4.6)	24.3 (4.9)	24.5 (4.7)	0.201
Satisfaction with family life (SWFaL) [Mean (SD)]^a^				
Father	24.7 (4.6)	24.6 (4.7)	24.7 (4.6)	0.686
Mother	24.0 (4.8)	23.7 (5.1)	23.8 (4.9)	0.610
Adolescent	25.3 (4.3)	24.7 (4.6)	25.0 (4.5)	0.051
Satisfaction with food-related life (SWFoL) [Mean (*SD*)]^a^				
Father	23.0 (4.7)	23.1 (4.3)	23.1 (4.5)	0.634
Mother	22.0 (4.5)	22.1 (4.5)	22.1 (4.5)	0.780
Adolescent	23.9 (4.6)	23.9 (4.4)	23.9 (4.5)	0.861
Job satisfaction (OJSS) [Mean (SD)]^a^				
Father	22.4 (4.4)	21.8 (4.5)	22.1 (4.8)	0.032
Mother	22.5 (4.8)	22.1 (4.8)	22.3 (4.8)	0.150
Adapted healthy eating index (AHEI) [Mean (SD)]^a^				
Father	61.5 (13.4)	60.9 (14.1)	61.2 (13.7)	0.483
Mother	65.6 (12.7)	65.7 (12.5)	65.3 (12.6)	0.579
Adolescent	65.7 (12.9)	65.3 (13.4)	65.5 (13.2)	0.635
Mothers' type of employment (%)^b^				
Employee	68.1	62.8	65.5	0.099
Self-employed	31.9	37.2	34.5	
Fathers' type of employment (%)^b^				
Employee	73.5	75.3	74.4	0.532
Self-employed	26.5	24.7	25.6	
Mothers' working hours (%)^b^				
45 h per week	48.4	44.0	46.2	0.194
< 45 h per week	51.6	56.0	53.8	
Fathers' working hours (%)^*b*^				
Man working 45 h per week	69.8	67.2	68.5	0.419
Man working < 45 h per week	30.2	32.8	30.2	

a Independent sample t-test.

b P-value corresponds to the (bilateral) asymptotic significance obtained in Pearson's Chi-square Test.

### 2.2. Measures

The following scales were answered by parents:

#### Overall Job Satisfaction Scale (OJSS)

Agho et al. (16) measured job satisfaction with a six-item scale (e.g., “I find real enjoyment in my job”). Responses were rated on a 5-point Likert scale, from 1 (completely disagree) to 5 (completely agree), and scores were obtained from the sum of the scores from the six items. This study used the validated Spanish version of the OJSS (36). In this study, the OJSS showed good internal reliability, with Omega coefficients for mothers ω = 0.92, and for fathers ω = 0.90. All standardized factor loadings were statistically significant (*p* < 0.001), ranging 0.581–0.945 for mothers, and 0.550–0.949 for fathers. The average extracted variance (AVE) values were higher than 0.50 for mothers (0.65), and fathers (0.61).

The three family members answered the following scales and instruments:

#### Satisfaction with Life Scale (SWLS)

Diener et al. (12) proposed this unidimensional five-item scale to evaluate individuals' overall cognitive judgments about their life (e.g.: “In most ways my life is close to my ideal”). Responses were rated on a 6-point Likert scale, from 1 (completely disagree) to 6 (completely agree), and scores were obtained from the sum of the scores from the five items. The validated Spanish version of the SWLS was used (75). The SWLS showed good internal reliability in this study, with Omega coefficients for mothers ω = 0.94, for fathers ω = 0.95, and for adolescents ω = 0.95. The SWLS standardized factor loadings were statistically significant (*p* < 0.001) and ranged from 0.798–0.919 for mothers, from 0.783–0.947 for fathers and from 0.736–0.888 for adolescents. The AVE values were higher than 0.50 for mothers (0.76), fathers (0.79), and adolescents (0.66).

#### Satisfaction with Family Life (SWFaL)

Zabriskie and McCormick (15) proposed this adaptation of the SWLS (12) by replacing the word “life” in the original items with “family life”. Responses were rated on a 6-point Likert scale, from 1 (completely disagree) to 6 (completely agree), and scores were obtained from the sum of the scores from the five items. The validated Spanish version of the SWFaL was used (76). The SWFaL showed good internal reliability in this study, with Omega coefficients for mothers ω = 0.91, for fathers ω = 0.91, and for adolescents ω = 0.90. The standardized factor loadings of the SWFaL were statistically significant (*p* < 0.001), ranging from 0.707–0.857 for mothers, from 0.707–0.892 for fathers and from 0.707–0.875 for adolescents. The AVE values were higher than 0.50 for mothers (0.67), fathers (0.68), and adolescents (0.65).

#### Satisfaction with Food-related Life (SWFoL)

Grunert et al. (17) designed this five-item scale that evaluates a person's overall assessment of their food and eating habits (e.g., “Food and meals are positive elements”). Responses were rated on a 6-point Likert scale, from 1 (completely disagree) to 6 (completely agree), and scores were obtained from the sum of the scores from the five items. The Spanish version of the SWFoL was used (75). The SWFoL showed good internal reliability in this study, with Omega coefficients for mothers ω = 0.89, for fathers ω = 0.89, and for adolescents ω = 0.88. The SWFoL standardized factor loadings ranged from 0.672 to 0.919 for mothers, from 0.660 to 0.922 for fathers and from 0.704 to 0.884 for adolescents, all statistically significant (p < 0.001). The AVE values were higher than 0.50 for mothers (0.63), fathers (0.63), and adolescents (0.60).

The above scales in their Spanish version have been validated in diverse Chilean samples. The OJSS has shown good internal consistency in workers (33, 36, 48). The SWLS, SWFoL and SWFaL have also shown good internal consistency in adult and adolescent samples (24, 49, 70, 76). The discriminant validity of the OJSS SWLS, SWFoL and SWFaL has been demonstrated in workers (36), and that of the SWLS, SWFoL and SWFaL has been shown in adult and adolescent samples (47, 48).

#### Adapted Healthy Eating Index (AHEI)

The AHEI comprises nine food groups and diet variety. This instrument is a version of the US Healthy Eating Index (77) adapted to Spanish by Norte and Ortiz (78). Each variable received a score from 0 to 10 according to the degree of compliance with dietary recommendations [see (78)]. The variable of diet variety is calculated based on the consumption frequency of the nine target foods. Respondents receive two points if they comply with each of the daily recommendations, and one point if they comply with each of the weekly recommendations. The AHEI score was calculated by summing the score from each variable, with a maximum score of 100 points. Scores above 80 indicate a “healthy” diet; scores between 51 and 80 indicate a diet that “requires changes”; and scores below 50 are categorized as “unhealthy” diets (77).

Lastly, the three family members answered *ad hoc* questions for sociodemographic and eating habits characterization. The three family members reported their age; adolescents reported their gender. Parents indicated their type of employment (“What is your type of job? Employee, self-employed”) and the number of working hours per week (“Do you work… Full-time, part-time, less than part-time”). Mothers reported the number of family members (“How many people live in your household, including yourself?”), the number of children (“How many of your children live with you?”), and the number of days per week that all family members eat together (“Indicate how many times in the last seven days your family has gathered for each meal: Breakfast, lunch and supper”). Questions about meals replaced dinner with “supper” because the latter is more customary in Chile. Total household income and its size allowed to determine the family socioeconomic status [SES, (97)].

### 2.3. Procedure

The call for participants was distributed *via* schools serving different socioeconomical backgrounds in Temuco and Rancagua. Parents received an invitation to participate in this study, and trained interviewers informed them of the study's aims and questionnaire topics, and the anonymous and confidential nature of their responses. Given the COVID-19 pandemic mandatory lockdowns in these cities throughout 2020 (Rancagua during June and July 2020, and Temuco during November and December 2020), data collection was conducted online between March and July 2020 in Rancagua, and between August and December 2020 in Temuco. Families whose three members (i.e., mother, father, and adolescent) agreed to participant were assigned an interviewer to oversee their participation. The response rate was 75.8% in Rancagua and 90.9% in Temuco, which resulted in the total sample of 860 families. Twenty-three families were discarded from the study in Rancagua because not all family members answered the corresponding questionnaire (mainly fathers). Interviewers established communication *via* telephone and email with one family member, most frequently the mother. This family member received the link to the three questionnaires, one for each family member. Each family was assigned an ID to identify its members in the databases. Upon completion of the three questionnaires, families received a gift card worth approximately 15 USD.

A pilot study was conducted in Temuco with fifty families. The same recruitment method and data collection procedure were followed, without any changes required. This study was approved by the Ethic Committee of Universidad de La Frontera (protocol 007/19).

### 2.4. Data analysis

For descriptive analyses, SPSS v.23 was used. The first seven hypotheses were tested using structural equation modeling (SEM) to assess the actor-partner interdependence model [APIM, (34)] with distinguishable dyads. To identify the effects among family members, APIM dyadic associations were tested within a mother-father-adolescent triadic design. This approach allows to assess the extent to which family members influence one another (79). Spillover (actor effects) are observed when characteristics are significant predictors of outcomes for an individual (i.e., parents' effects of OJSS, SWFaL and SWFoL on their own SWLS; adolescents' effects of their SWFaL and SWFoL on their own SWLS). Crossovers (partner effects) are observed when one family member's characteristics influence another family member's outcome (i.e., the effect of mothers' OJSS, SWFaL and SWFoL on fathers' SWLS and vice versa; each parent's OJSS, SWFaL and SWFoL on adolescents' SWLS; adolescents' SWFaL and SWFoL on their parents' SWLS).

The influence of one family member's satisfaction on that of another member is controlled in the APIM by correlating the independent variables of each dyad member (i.e., fathers' and mothers' OJSS, the three family members' SWFaL and SWFoL). Correlations between the residual errors of the dependent variables of each dyad member (i.e., the three family members' SWLS) are also examined in the APIM to control for other sources of interdependence between partners (34).

SEM was conducted using MPlus 8.5. The SEM parameters were estimated *via* robust unweighted least squares (ULSMV), and a polychoric correlation matrix was used given the ordinal scale of the items. The model fit of the data were determined with the Tucker-Lewis index (TLI) and the comparative fit index (CFI) with a cutoff value of 0.90 for an acceptable fit. Both the TLI and the CFI had a good fit with values above 0.95. The root mean square error of approximation (RMSEA) was also considered to measure poorness-of-fit. RMSEA values lower than 0.06 indicate a good fit, and values below 0.08 indicate an acceptable fit (80).

For hypotheses 8–11, we tested differences between mothers' and fathers' path coefficients *via* SEM. That is, we explored gender differences in parents in their spillover (each parent's OJJS, SWFaL, SWFoL on their own life satisfaction) and crossover effects (each parent's OJJS, SWFaL, SWFoL on their partner's life satisfaction, each parent's OJJS, SWFaL, SWFoL on the adolescent's life satisfaction). Lastly, we tested moderating effects proposed in RQ1 through multi-group SEM (81), comparing direct effect parameters between groups (defined by dichotomous moderators) for each model path. We established evidence of the moderation effect when a direct estimate in the model had a statistical difference between groups.

## 3. Results

### 3.1. APIM results: Testing spillover-crossover hypotheses

Table 2 shows the correlations for job satisfaction (JS), family life satisfaction (SWFaL), food-related life satisfaction (SWFoL) and life satisfaction (LS). Most of the correlations were significant and in the expected directions, except the correlation between mothers' JS and adolescents' SWFoL. Correlation values between the three family members' SWFaL and SWLS were of high strength. However, the value of the squared correlation between mothers' SWFaL and SWLS (0.41) was lower than the AVE of the scales (0.67 and 0.76, respectively), which verified the discriminant validity between the SWFaL and SWLS in the mothers subsample. Similarly, the value of the squared correlation between fathers' SWFaL and SWLS (0.50) was lower than the AVE of the scales (0.68 and 0.79, respectively), while the value of the squared correlation between adolescents' SWFaL and SWLS (0.52) was lower than the AVE of the scales (0.65 and 0.66, respectively). Therefore, the discriminant validity between the SWFaL and SWLS was also verified in the fathers and adolescents subsamples.

**Table 2:** Correlations between the Overall Job Satisfaction Scale (OJSS), Satisfaction with Family life scale (SWFaL), Satisfaction with Food-related Life scale (SWFoL), and Satisfaction with Life Scale (SWLS) for each family member.

**Scale**	**1**	**2**	**3**	**4**	**5**	**6**	**7**	**8**	**9**	**10**	**11**
1. Mothers' OJJS	-	0.274^***^	0.204^***^	0.134^***^	0.073^*^	0.148^***^	0.109^**^	0.062	0.243^***^	0.152^***^	0.070^*^
2. Fathers' OJJS		-	0.147^***^	0.246^***^	0.128^***^	0.105^**^	0.244^***^	0.093^**^	0.224^***^	0.328^***^	0.152^***^
3. Mothers' SWFaL			-	0.432^***^	0.243^***^	0.368^***^	0.206^***^	0.221^***^	0.645^***^	0.405^***^	0.202^***^
4. Fathers' SWFaL				-	0.337^***^	0.250^***^	0.334^***^	0.222^***^	0.453^***^	0.706^***^	0.301^***^
5. Adolescents' SWFaL					-	0.159^***^	0.240^***^	0.413^***^	0.336^***^	0.312^***^	0.719^***^
6. Mothers' SWFoL						-	0.360^***^	0.293^***^	0.376^***^	0.244^***^	0.138^***^
7. Fathers' SWFoL							-	0.376^***^	0.253^***^	0.368^***^	0.206^***^
8. Adolescents' SWFoL								-	0.219^***^	0.188^***^	0.439^***^
9. Mothers' SWLS									-	0.486^***^	0.309^***^
10. Fathers' SWLS										-	0.276^***^
11. Adolescents' SWLS											-

* p < 0.05,

** p < 0.01,

*** p < 0.001.

**Figure 1:**
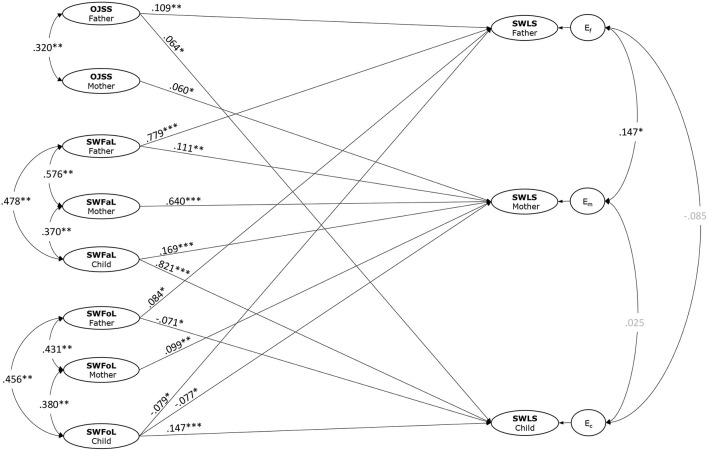
Actor-partner interdependence model of the effect of Overall Job Satisfaction Scale (OJSS), Satisfaction with Family Life (SWFaL), Satisfaction with Food-related Life (SWFoL) on Satisfaction with Life (SWLS) in dual-earner couples with adolescent children. E_f_, E_m_, and E_c_: residual errors on SWLS for fathers, mothers and adolescent children, respectively. Only significant path coefficients are shown *p < 0.05, **p < 0.01, ***p < 0.001.

The model that assessed the APIM association between both parents' JS and the three family members' SWFaL, SWFoL and LS showed a good fit to the data (RMSEA = 0.032; CFI = 0.957; TLI = 0.953). As shown in Figure 1, we found significant correlations (covariances) for parents' JS (r = 0.320, *p* < 0.001), SWFaL (r = 0.576, *p* < 0.001), and SWFoL (r = 0.431, *p* < 0.001). We also found significant correlations for mothers' and adolescents' SWFaL (r = 0.370, *p* < 0.001) and SWFoL (r = 0.380, *p* < 0.000), and for fathers' and adolescents' SWFaL (r = 0.478, *p* < 0.001) and SWFoL (r = 0.456, *p* < 0.001). The correlation between the residual errors of both parents' LS was significant (r = 0.147, p < 0.004), while the correlation between the residual errors of mothers' and adolescents' LS (r = 0.025, *p* = 0.682) and between the residual errors of fathers' and adolescents' LS (r = −0.085, *p* = 0.240) were not significant.

Figure 1 displays the estimation of the structural model. Hypothesis 1 examined spillover associations for fathers. The path coefficients indicated that fathers' LS was positively associated with their own JS (H1a, γ = 0.109, *p* < 0.001), SWFaL (H1b, γ = 0.779, p < 0.001) and SWFoL (H1c, γ = 0.084, *p* = 0.020). Hypothesis 2 tested spillover associations for mothers. Path coefficients indicated that mothers' LS was positively associated with their own JS (H2a, γ = 0.060, *p* = 0.021), SWFaL (H2b, γ = 0.640, *p* < 0.001) and SWFoL (H2c, γ = 0.099, *p* = 0.002). Likewise, Hypothesis 3 tested spillover associations for adolescents. Path coefficients indicated that adolescents' LS was positively associated with their own SWFaL (H3a, γ = 0.821, *p* < 0.001) and SWFoL (H3b, γ = 0.147, *p* < 0.001). Hence, Hypotheses 1 to 3 were supported.

Hypothesis 4 to 6 examined crossover associations. Hypothesis 4 posed positive crossover effects with fathers' LS as the outcome. Results showed that mothers' JS (H4a, γ = −0.005, p = 0.840), SWFaL (H4b, γ = 0.031, p = 0.345) and SWFoL (H4c, γ = −0.004, p = 0.886) were not significantly associated with the fathers' LS. Likewise, adolescents' SWFaL (H4d, γ = 0.051, p = 0.111) was not significantly associated with fathers' LS, while adolescents' SWFoL (H4e, γ = −0.079, p = 0.011) was negatively associated with fathers' LS. These findings thus did not support Hypothesis 4.

Hypothesis 5 posed positive crossover effects with mothers' life satisfaction as the outcome. Fathers' JS (H5a, γ = 0.056, *p* = 0.068), and SWFoL (H5c, γ = −0.006, *p* = 0.860) were not significantly associated with mothers' LS. By contrast, fathers' SWFaL (H5b, γ = 0.111, *p* = 0.007) was positively associated with mothers' LS. Adolescents' SWFaL (H5d, γ = 0.111, *p* = 0.007) was positively associated with mothers' LS, while adolescents' SWFoL (H5e, γ = −0.077, *p* = 0.025) was negatively associated with mother's LS. These findings supported only H5b and H5d.

Lastly, Hypothesis 6 posed positive crossover effects with adolescents' LS as the outcome. Fathers' JS was positively associated with adolescents' LS (H6a, γ = 0.064, *p* = 0.028). By contrast, fathers' SWFoL was negatively associated with adolescents' LS (H6c, γ = −0.071, *p* = 0.020), and fathers' SWFaL was not associated with adolescents' LS (H6b, γ =0.020, *p* =0.613). Mothers' JS (H6d, γ =0.002, *p* =0.919), SWFaL (H6e, γ = −0.028, *p* = 0.373) and SWFoL (H6f, γ = −0.017, *p* = 0.567) were not statistically associated with adolescents' LS. These findings thus only supported H6a.

### 3.2. Testing gender differences in spillover and crossover effects

Hypotheses 7 to 9 proposed gender differences in spillover and crossover effects for mother-father dyads (Table 3). First, the association between fathers' JS and LS (spillover) was significantly higher than the association between mothers' JS and fathers' LS (crossover; *p* = 0.002). By contrast, the association between the mothers' JS and LS (spillover) did not differ from the association between the fathers' JS and the mothers' LS (crossover) (*p* = 0.819). These findings supported H7a and H7b. Next, the association between fathers' SWFaL and LS (spillover) was significantly higher than the association between mothers' SWFaL and fathers' LS (crossover; *p* < 0.001). The association between mothers' SWFaL and LS (spillover) was significantly higher than the association between fathers' SWFaL and mothers' LS (crossover; *p* < 0.001). These findings supported H8a and H8b. Lastly, the association between mothers' SWFoL and LS (spillover) was significantly higher than the association between fathers' SWFoL and mothers' LS (crossover; *p* = 0.002). A similar result was obtained in the opposite way (*p* = 0.021). Thus, these findings supported H9a, but not H9b.

**Table 3:** Estimates for structural coefficients and moderation role of the parents' gender in the model that explains the relationships between Overall Job Satisfaction Scale (OJSS), Satisfaction with Family Life (SWFaL), and Satisfaction with Food-related Life (SWFoL) on Satisfaction with Life (SWLS) in dual-earner couples with adolescent children.

**Structural path and direction**	**Estimate**	***p*–value**	**Structural path and direction**	**Estimate**	***p–*value**	***p*–value for estimate differences**
Mother's OJSS → mother's SWLS	0.066	0.009	Father's OJSS → mother's SWLS	0.056	0.057	0.819
Mother's SWFaL → mother's SWLS	0.618	0.000	Father's SWFaL → mother's SWLS	0.119	0.001	0.000
Mother's SWFoL → mother's SWLS	0.110	0.000	Father's SWFoL → mother's SWLS	−0.005	0.865	0.022
Mother's OJSS → father's SWLS	−0.006	0.803	Father's OJSS → father's SWLS	0.120	0.000	0.002
Mother's SWFaL → father's SWLS	0.056	0.046	Father's SWFaL → father's SWLS	0.728	0.000	0.000
Mother's SWFoL → father's SWLS	−0.005	0.830	Father's SWFoL → father's SWLS	0.109	0.001	0.021
Mother's OJSS → adolescent's SWLS	0.007	0.764	Father's OJSS → adolescent's SWLS	0.055	0.045	0.237
Mother's SWFaL → adolescent's SWLS	−0.016	0.559	Father's SWFaL → adolescent's SWLS	0.028	0.396	0.367
Mother's SWFoL → adolescent's SWLS	−0.024	0.386	Father's SWFoL → adolescent's SWLS	−0.058	0.042	0.460

Hypotheses 10 and 11 proposed crossover effects for both mother-adolescent and father-adolescent dyads. No significant differences were found while comparing the association between mothers' and fathers' JS and adolescents' LS (*p* = 0.237), between mothers' and fathers' SWFaL and adolescents' LS (*p* = 0.347), nor between mothers' and fathers' SWFoL and adolescents' LS (*p* = 0.460). Thus, H10 and H11 were not supported.

### 3.3. The moderating role of parents' type of employment and working hours

Using multi-group analyses, parents' type of employment and working hours were examined as categorical variables, that is, respectively, employee vs. self-employed and 45 h/week vs. <45 h/week. We first address type of employment. For mothers, the analysis showed a good fit to the data (*RMSEA* = 0.039, *CFI* =0.974, *TLI* = 0.974). Mothers' type of employment moderated the association between both parents' SWFaL and fathers' LS (γ = −0.496, *p* = 0.037). In families with employed mothers (Table 4), mothers' SWFaL was not statistically associated with fathers' LS (γ =0.006, *p* = 0.851), while fathers' SWFaL was positively associated with their own LS (γ =0.783 *p* < 0.001). By contrast, in families with self-employed mothers, both mothers' (γ =0.148, *p* = 0.001) and fathers' (γ =0.687, *p* < 0.001). SWFaL was significantly associated with fathers' LS. Mothers' type of employment also moderated the relationship between both parents' SWFoL and fathers' LS (γ = 0.847, *p* = 0.001). In families with employed mothers, the association between mothers' SWFoL was not associated with the fathers' LS (γ = 0.026, *p* = 0.182), while fathers' SWFoL was positively associated with their own LS (γ = 0.068, *p* = 0.039). By contrast, in families with self-employed mothers, both mothers' (γ = 0.126, *p* = 0.061) and fathers' (γ = 0.297, *p* < 0.001). SWFoL was significantly related to fathers' LS.

**Table 4:** Estimates for structural coefficients and moderation role of the mother's type of employment in the model that explains the relationships between Overall Job Satisfaction Scale (OJSS), Satisfaction with Family Life (SWFaL), and Satisfaction with Food-related Life (SWFoL) on Satisfaction with Life (SWLS) in dual-earner couples with adolescent children.

**Structural path and direction**	**Employee**		**Self-Employee**		**Structural path and direction**	**Employee**		**Self-Employee**		***P*-value for estimate differences**
	**Estimate**	***p***	**Estimate**	***p***		**Estimate**	***p***	**Estimate**	***p***	
M OJSS → M SWLS	0.100	0.001	0.012	0.769	F OJSS → M SWLS	0.059	0.091	0.041	0.374	0.433
M SWFaL → M SWLS	0.640	0.000	0.572	0.000	F SWFaL → M SWLS	0.092	0.038	0.191	0.000	0.132
M SWFoL → M SWLS	0.092	0.006	0.160	0.002	F SWFoL → M SWLS	0.002	0.966	−0.022	0.696	0.386
M OJSS → F SWLS	0.033	0.278	−0.046	0.330	F OJSS → F SWLS	0.115	0.000	0.113	0.016	0.372
M SWFaL → F SWLS	0.006	0.851	0.148	0.001	F SWFaL → F SWLS	0.783	0.000	0.687	0.000	0.037^*^
M SWFoL → F SWLS	0.026	0.182	0.126	0.006	F SWFoL → F SWLS	0.068	0.039	0.297	0.000	0.001^**^
M OJSS → A SWLS	−0.005	0.868	0.019	0.615	F OJSS → A SWLS	0.059	0.095	0.057	0.149	0.732
M SWFaL → A SWLS	−0.008	0.793	−0.038	0.459	F SWFaL → A SWLS	0.065	0.141	0.037	0.405	0.992
M SWFoL → A SWLS	−0.024	0.470	−0.050	0.248	F SWFoL → A SWLS	−0.066	0.076	−0.026	0.590	0.492

**#x0002A;:** p < 0.05,

** p < 0.01.

M, Mother; F, Father; A, Adolescent.

For fathers, the multi-group analysis also showed a good fit to the data (RMSEA = 0.039; CFI = 0.974; TLI = 0.973). Fathers' type of employment moderated the association between both parents' JS and mothers' LS (γ = 0.536, *p* = 0.016). In families with employed fathers (Table 5), mothers' JS was positively associated with their own LS (γ = 0.090, *p* < 0.001), while fathers' JS was not statistically associated with mothers' LS (γ = 0.034, *p* = 0.298). In families with self-employed fathers, mothers' JS was not significantly associated with their own LS (γ = −0.066, *p* = 0.257), while fathers' JS was positively associated with mothers' LS (γ = 0.167 *p* = 0.011). Fathers' type of employment also moderated the association between both parents' SWFoL and mothers' LS (γ = −0.735, *p* = 0.001). In families with employed and self-employed fathers, mothers' SWFoL was significantly associated with their own LS although by a stronger path coefficient in families with self-employed fathers (employed fathers γ =0.070, *p* = 0.018, self-employed fathers γ = 0.350, p < 0.001). Fathers' SWFoL was not statistically related to mothers' LS (employed fathers γ = 0.005, *p* = 0.880, self-employed fathers γ = −111, *p* = 0.054).

**Table 5:** Estimates for structural coefficients and moderation role of the father's type of employment in the model that explains the relationships between Overall Job Satisfaction Scale (OJSS), Satisfaction with Family Life (SWFaL), and Satisfaction with Food-related Life (SWFoL) on Satisfaction with Life (SWLS) in dual-earner couples with adolescent children.

**Structural path and direction**	**Employee**		**Self-Employee**		**Structural path and direction**	**Employee**		**Self-Employee**		***P*-value for estimate differences**
	**Estimate**	***p***	**Estimate**	***p***		**Estimate**	***p***	**Estimate**	***p***	
M OJSS → M SWLS	0.090	0.000	−0.066	0.257	F OJSS → M SWLS	0.034	0.298	0.167	0.011	0.016^*^
M SWFaL → M SWLS	0.645	0.000	0.445	0.000	F SWFaL → M SWLS	0.136	0.000	0.179	0.019	0.102
M SWFoL → M SWLS	0.070	0.018	0.350	0.000	F SWFoL → M SWLS	0.005	0.880	−0.111	0.054	0.001^**^
M OJSS → F SWLS	0.005	0.862	−0.062	0.138	F OJSS → F SWLS	0.118	0.000	0.153	0.001	0.202
M SWFaL → F SWLS	0.053	0.102	0.106	0.057	F SWFaL → F SWLS	0.714	0.000	0.754	0.000	0.786
M SWFoL → F SWLS	0.006	0.851	−0.022	0.618	F SWFoL → F SWLS	0.176	0.000	−0.043	0.379	0.064
M OJSS → A SWLS	0.006	0.797	0.001	0.983	F OJSS → A SWLS	0.051	0.084	0.056	0.299	0.895
M SWFaL → A SWLS	0.011	0.711	−0.046	0.129	F SWFaL → A SWLS	0.011	0.773	0.124	0.062	0.105
M SWFoL → A SWLS	−0.026	0.437	0.020	0.704	F SWFoL → A SWLS	−0.073	0.026	−0.037	0.470	0.907

* p < 0.05,

** p < 0.01.

M, Mother; F, Father; A, Adolescent.

Regarding working hours, for mothers, the multi-group analysis showed a good fit to the data (RMSEA = 0.042, CFI = 0.971, TLI = 0.970). Mothers' working hours moderated the association between both parents' JS and fathers' LS (γ = 0.361, *p* = 0.027). In families with mothers working 45 h/week (Table 6), the association between mothers' JS was not statistically associated with fathers' LS (γ = 0.013, *p* = 0.692), nor was there an association between fathers' JS and their own LS (γ = 0.046 *p* = 0.280). Similarly, in families with mothers working <45 h per week, the association between mothers' JS and fathers' LS was not statistically significant (γ = −0.022 *p* = 0.514). However, the association between fathers' JS and their own LS was positive and significant (γ = 0.194 *p* < 0.001). Mother's working hours also moderated the association between both parents' JS and adolescents' LS (γ = 0.370, *p* = 0.030). In families with mother working 45 h per week neither mothers' (γ = 0.035, *p* = 0.263) nor fathers' (γ = 0.000 *p* = 0.994). JS were significantly associated with adolescents' LS. In families with mothers working < 45 h/week, mothers' JS was not significantly associated with adolescents' LS (γ = −0.027 *p* = 0.410). However, in this case fathers' JS was positively related to adolescents' LS (γ = 0.114 *p* = 0.001).

**Table 6:** Estimates for structural coefficients and moderation role of the mother's working hours in the model that explains the relationships between Overall Job Satisfaction Scale (OJSS), Satisfaction with Family Life (SWFaL), and Satisfaction with Food-related Life (SWFoL) on Satisfaction with Life (SWLS) in dual-earner couples with adolescent children.

**Structural path and direction**	**45 h/week**		<**45 h/week**		**Structural path and direction**	**45 h/week**		<**45 h/week**		***P*-value for estimate differences**
	**Estimate**	***p***	**Estimate**	***p***		**Estimate**	***p***	**Estimate**	***p***	
M OJSS → M SWLS	0.086	0.012	0.057	0.105	F OJSS → M SWLS	0.062	0.167	0.052	0.112	0.830
M SWFaL → M SWLS	0.619	0.000	0.639	0.000	F SWFaL → M SWLS	0.070	0.206	0.136	0.000	0.900
M SWFoL → M SWLS	0.071	0.087	0.162	0.000	F SWFoL → M SWLS	0.024	0.673	−0.025	0.431	0.138
M OJSS → F SWLS	0.013	0.692	−0.022	0.514	F OJSS → F SWLS	0.046	0.280	0.194	0.000	0.027^*^
M SWFaL → F SWLS	0.057	0.123	0.097	0.012	F SWFaL → F SWLS	0.761	0.000	0.663	0.000	0.146
M SWFoL → F SWLS	−0.014	0.692	−0.011	0.724	F SWFoL → F SWLS	0.141	0.004	0.093	0.006	0.548
M OJSS → A SWLS	0.035	0.263	−0.027	0.410	F OJSS → A SWLS	0.000	0.994	0.114	0.001	0.030^*^
M SWFaL → A SWLS	−0.019	0.562	−0.005	0.897	F SWFaL → A SWLS	0.064	0.169	−0.001	0.973	0.275
M SWFoL → A SWLS	−0.035	0.321	−0.004	0.917	F SWFoL → A SWLS	−0.009	0.833	−0.135	0.001	0.101

* p < 0.05.

M, Mother; F, Father; A, Adolescent.

For fathers, the multi-group analysis showed a good fit to the data (RMSEA = 0.040, CFI = 0.974, TLI = 0.973). However, the fathers' working did not moderate any of the relationships tested (Table 7).

**Table 7:** Estimates for structural coefficients and moderation role of the father's working hours in the model that explains the relationships between Overall Job Satisfaction Scale (OJSS), Satisfaction with Family Life (SWFaL), and Satisfaction with Food-related Life (SWFoL) on Satisfaction with Life (SWLS) in dual-earner couples with adolescent children.

**Structural path and direction**	**45 h/week**		<**45 h/week**		**Structural path and direction**	**45 h/week**		<**45 h/week**		***P*-value for estimate differences**
	**Estimate**	***p***	**Estimate**	***p***		**Estimate**	***p***	**Estimate**	***p***	
M OJSS → M SWLS	0.072	0.008	0.084	0.086	F OJSS → M SWLS	0.063	0.060	0.012	0.820	0.541
M SWFaL → M SWLS	0.630	0.000	0.568	0.000	F SWFaL → M SWLS	0.149	0.000	0.062	0.300	0.960
M SWFoL → M SWLS	0.089	0.004	0.152	0.010	F SWFoL → M SWLS	−0.018	0.642	0.078	0.124	0.735
M OJSS → F SWLS	0.008	0.805	−0.004	0.920	F OJSS → F SWLS	0.138	0.000	0.048	0.308	0.337
M SWFaL → F SWLS	0.028	0.392	0.066	0.061	F SWFaL → F SWLS	0.744	0.000	0.647	0.000	0.245
M SWFoL → F SWLS	−0.007	0.814	−0.060	0.218	F SWFoL → F SWLS	0.094	0.003	0.196	0.006	0.198
M OJSS → A SWLS	0.024	0.383	−0.019	0.626	F OJSS → A SWLS	0.086	0.007	−0.045	0.390	0.352
M SWFaL → A SWLS	−0.022	0.451	0.043	0.391	F SWFaL → A SWLS	0.027	0.539	0.028	0.505	0.550
M SWFoL → A SWLS	−0.012	0.725	−0.094	0.52	F SWFoL → A SWLS	−0.066	0.066	−0.042	0.333	0.286

M, Mother; F, Father; A, Adolescent.

### 3.4. The moderating role of city of residence

Using multi-group analyses, the city of residence was examined as a categorical variable (Rancagua vs. Temuco). The multi-group analysis had fit indices that showed a good fit with the data (RMSEA = 0.037, CFI = 0.978, TLI = 0.978). The city of residence moderated the association between both parent's family life satisfaction and the father's life satisfaction (γ = −0.846, *p* < 0.001). In families living in Rancagua (Table 8), the association between the mothers' family satisfaction was not statistically associated with the fathers' life satisfaction (γ = −0.004, *p* = 0.001), while the fathers' family life satisfaction was positively associated with their own life satisfaction (γ = 0.814 *p* < 0.001). By contrast, in families living in Temuco, both the mothers' (γ = 0.132, *p* = 0.001) and the fathers' (γ = 0.625, *p* < 0.001) family life satisfaction were significantly associated with the fathers' life satisfaction. The city of residence also moderated the relationship between both parents' job satisfaction and the adolescents' life satisfaction (γ = −0.600, *p* < 0.001). In families living in Rancagua, neither the mothers' (γ = −0.035, *p* = 0.273) nor the fathers' (γ = −0.034, *p* = 0.358) job satisfaction were statistically associated with the adolescents' life satisfaction. By contrast, in families living in Temuco, the association between mothers' job satisfaction was not statistically associated with the adolescents' life satisfaction (γ = 0.053, *p* = 0.082), while the fathers' job satisfaction was positively associated with the adolescents' life satisfaction (γ = 0.163, *p* < 0.001).

**Table 8:** Estimates for structural coefficients and moderation role of the city of residence in the model that explains the relationships between Overall Job Satisfaction Scale (OJSS), Satisfaction with Family Life (SWFaL), and Satisfaction with Food-related Life (SWFoL) on Satisfaction with Life (SWLS) in dual-earner couples with adolescent children.

**Structural path and direction**	**Rancagua**		**Temuco**		**Structural path and direction**	**Rancagua**		**Temuco**		***P*-value for estimate differences**
	**Estimate**	***p***	**Estimate**	***p***		**Estimate**	***p***	**Estimate**	***p***	
M OJSS → M SWLS	0.073	0.008	0.067	0.118	F OJSS → M SWLS	0.080	0.048	0.044	0.274	0.737
M SWFaL → M SWLS	0.553	0.000	0.647	0.000	F SWFaL → M SWLS	0.150	0.004	0.105	0.008	0.183
M SWFoL → M SWLS	0.138	0.000	0.101	0.042	F SWFoL → M SWLS	−0.003	0.951	−0.016	0.713	0.822
M OJSS → F SWLS	−0.075	0.017	0.067	0.061	F OJSS → F SWLS	0.101	0.002	0.123	0.001	0.082
M SWFaL → F SWLS	−0.004	0.895	0.132	0.001	F SWFaL → F SWLS	0.814	0.000	0.625	0.000	0.000^***^
M SWFoL → F SWLS	−0.016	0.579	−0.009	0.816	F SWFoL → F SWLS	0.118	0.007	0.097	0.022	0.624
M OJSS → A SWLS	−0.035	0.273	0.053	0.082	F OJSS → A SWLS	−0.034	0.358	0.163	0.000	0.000^***^
M SWFaL → A SWLS	−0.026	0.519	−0.038	0.193	F SWFaL → A SWLS	−0.020	0.717	0.061	0.074	0.145
M SWFoL → A SWLS	−0.026	0.519	−0.040	0.250	F SWFoL → A SWLS	−0.053	0.163	−0.071	0.068	0.971

*** p < 0.001.

M, Mother; F, Father; A, Adolescent.

## 4. Discussion

Our results support spillover and crossover effects for domain and life satisfaction between parents, their partner, and their adolescent child. Findings show that both parents' life satisfaction (LS) is positively associated with their own job (JS), family life (SWFaL) and food-related life (SWFoL) satisfaction, while adolescents' LS is positively related to their own SWFaL and SWFoL. These findings are consistent with the bottom-up theoretical approach to life satisfaction (11). In line with family systems theory (21), we also found that one parent's LS is associated with the other parent's domain satisfaction, but also that each parent's LS is associated with their adolescent children's domain satisfaction and vice versa.

Furthermore, we found positive and negative crossovers between domain satisfaction and LS between parents and adolescents. Between parents, fathers showed asymmetric positive crossovers to mothers between JS and LS, and between SWFaL and LS. There were no crossovers between SWFoL and LS in mother-father dyads. In mother-adolescent dyads, adolescents showed one asymmetrical positive crossover between their SWFaL and their mothers' LS, and one asymmetrical negative crossover between their SWFoL and their mothers' LS. In father-adolescent dyads, we found one symmetrical (i.e., reciprocal) negative crossover between SWFoL and LS. Fathers' JS also crossed to adolescents' LS. Therefore, as fathers', mothers', and adolescents' LS can be influenced by the other family members' domain satisfaction, interventions to improve LS should be carried out not individually, but in the family unit.

Lastly, we observed different gender patterns in spillover and crossover associations for JS. We also found that both parents' type of employment and mothers' working hours, and the city of residence moderated some spillover and crossover associations between domain and life satisfaction in the three family members. These findings are discussed in detail below.

### 4.1. Spillover effects

We found evidence for spillover effects for fathers (H1), mothers (H2), and adolescents (H3). Specifically, both parents showed a positive association between JS, SWFaL, SWFoL, and LS. These spillover effects align with, and expand on, findings from both before and during the pandemic. The three domains (job, family, and food) contribute to overall LS, but the strongest correlation in the three family members appeared between SWFaL and LS. Previous studies have indeed established that SWFaL is a stronger contributor to LS than JS for adults (36, 39, 49), and stronger than SWFoL for adults and adolescents (18, 47, 49, 76). It is also notable in our study that SWFaL is the main contributor to LS in both parents. Based on previous literature (57, 58), we expected that SWFaL would be a stronger contributor to women's LS than to men. However, other evidence (36, 63) also shows that the main determinants of LS relate to social relationships and may be similar for men and women. The importance of social resources as contributors to LS has increased during the COVID-19 pandemic (44), but these resources, namely family life, may be culture-sensitive (39, 82). In this regard, Chile is a relatively collectivistic culture that places great importance on family and on fostering positive interpersonal relationships (66). On this basis, the results of this study and previous ones suggest that the family domain is relevant for men and women in dual-earner couples in Chile regardless of the confinement context of the COVID-19 pandemic.

The relevance of the job and food domains as contributors to LS also appear to remain stable during the pandemic for men and women. Similar to pre-pandemic results (36), in our study the spillover association between SWFoL and LS was stronger than between JS and LS in mothers, while we found the opposite trend in fathers. These gendered patterns are supported by findings showing that women invest more time in the food domain that men. Studies conducted before (59) and during (83) the pandemic in Chile report that women spend significantly more hours per day cooking for the household than their male partners, under the socially shared assumption that cooking is women's responsibility. Moreover, both LS and SWFoL are linked to healthy diets (18, 28, 52, 53), and women have healthier diets than men [e.g., (76, 84, 85)], a result that we also observed in this study (see AHEI scores). Taken together, these results suggest that gendered roles in society and socialization practices may lead men and women to derive LS from different domains (63), even during the pandemic.

### 4.2. Crossover effects

We hypothesized crossover effects from mothers and adolescents to fathers' LS (H4). There is evidence of crossovers from women's job conditions to their male partner's LS in Germany (i.e., work-to-family conflict, 45), while this outcome was not significant in Chile for women's JS (36) and work-life balance (25). Our results align with the latter findings, as mothers' JS did not cross over to fathers' LS (H4a). The discrepancies between studies conducted in Germany and Chile may reflect gender inequalities associated to the culture or country. Out of 153 countries in the Global Gender Gap Index Ranking, Germany ranks in the 10th place while Chile ranks in the 57th (86). Traditional gendered socialization in Latin American countries, in keeping with gender role theory, characterizes women as responsible for household and family tasks regardless of their work outside the home (59), while men's work role outside the home is considered providing for the family (24). Furthermore, JS has been linked to income (33, 69, 87), and Schnettler et al. (36) suggests that, in Latin American cultures, it is likely that the family's larger income depends more on the man's than the woman's job. These factors help thus explain why mothers' JS does not cross over to fathers' LS.

Mothers' SWFaL did not cross over to fathers' LS (H4b), a result that may be related to pandemic-specific conditions. Positive crossover between SWFaL and LS in dual-earner couples is explained on the basis that couples share many significant experiences and daily domestic responsibilities (88, 89). In the context of COVID-19 pandemic, increased work and family demands (4, 90–92) may interfere with shared family-related experiences. Moreover, the literature is consistent in reporting that in dual-earner families, the extra family demands during the pandemic (e.g., childcare, home schooling, domestic chores) have fallen mostly on women (8, 83, 91, 92). Therefore, although mothers' SWFaL in this study is similar to that of pre-pandemic studies (36), we hypothesize that this lack of crossover entails that fathers did not account for mothers' family-related assessments, even if both parents were confined to shared home spaces for longer periods.

Mothers' SWFoL also did not cross over to fathers' LS (H4c). We expected a significant crossover given the evidence showing that families got together more frequently for meals during the COVID-19 pandemic (18, 20). Frequent family meals have been linked to greater family interaction and higher levels of SWFoL (76, 93). Nevertheless, although in this study the frequency of family meals was indeed higher than in pre-pandemic studies in Chile [e.g., (76)], this null finding suggests that this frequency alone does not contribute to direct crossovers between SWFoL and LS in dual-earner parents.

Crossover effects from adolescents' domain satisfaction to fathers' LS were mixed. Adolescents' SWFaL did not crossover to fathers' LS (H4d), in line with findings from a pre-pandemic study in Chile (59). On the other hand, also against expectations, there was a negative crossover association between adolescents' SWFoL and fathers' LS (H4e). This finding contradicts a pre-pandemic study in Chile that showed no significant crossover effects in the same variables for adolescent-father dyads (54). Therefore, this result may reflect a greater involvement of fathers in their children's eating habits during the pandemic, a trend consistently reported in the early stages of this public health crisis (18, 20, 28).

Next, we hypothesized crossover effects from fathers and adolescents to mothers' LS (H5). Fathers' JS did not cross over to mothers' LS (H5a), contrary to evidence showing crossover effects from men's job-related variables to women's LS (25, 36). One possible explanation for this null result might be that Chilean families with medium and low incomes received financial aid from the State and were allowed to withdraw part of their pension funds (94). This type of support may have lessened the importance that mothers attribute to the father's income, and in turn to JS, as discussed above.

On the other hand, fathers' SWFaL crossed over to the mothers' LS (H5b). This finding is in line with the higher likelihood of crossovers from men to women, that is, women's traditional socialization encourages them to be more sensitive than men to the feelings and emotions of their male partner (60, 62). However, this expectation was not met for another hypothesis of this study, as there were no crossover effects from fathers' SWFoL to mothers' LS. It may be possible that, given mothers' higher burden in multiple family and household-related tasks during the pandemic (8, 84, 92, 93), they could either not prioritize nor attend to their male partners' food-related life as traditional gender roles dictate, and thus this variable did not contribute to their LS.

Regarding crossover effects from adolescents' domain satisfaction to mothers' LS, findings show that the greater SWFaL in adolescents, the greater LS in their mothers (H5e). Therefore, public campaigns to promote healthy interactions and family functioning in families can not only enhance each family member's SWFaL and LS, but also mothers' LS. On the other hand, although there was a significant crossover from adolescents' SWFoL to mothers' LS, this relationship was negative (H5d). These findings contradict results from a pre-pandemic study showing a positive relationship between adolescents' SWFoL and mothers' LS (31). Although more research is required, this result may relate to pandemic conditions, similar to what was previously discussed for adolescents and fathers regarding a negative association between adolescents' food domain and their parents' LS. Therefore, campaigns promoting an adequate diet quality in adolescents may have a positive impact on their health and wellbeing, but it can also contribute to their parents' LS.

Lastly, we hypothesized crossover effects from fathers and mothers to adolescents' LS (H6). Fathers' JS (H6a) and SWFoL (H6c) crossed over to adolescents' LS, although the first crossover was positive and the second one, contrary to the expectation, was negative. By contrast, both parents' SWFaL, and mothers' JS and SWFoL did not cross over to adolescents' LS (H6b, H6d, H6e, and H6f). The positive association between fathers' JS and adolescents' LS and the lack of a significant association between mothers' JS and adolescents' LS reinforce the idea about the importance of the fathers' job for the family (24, 36). We hypothesize here an intragenerational transmission of gender roles in which adolescents are more sensitive to their fathers', rather than their mothers', job and how it contributes to their own living conditions. On the other hand, the negative association between fathers' SWFoL and adolescents' LS, and the lack of association between mothers' SWFoL and adolescents' LS, may be explained by both parents' diet quality (see AHEI scores). Fathers' SWFoL may negatively influences adolescents' LS because the latter perceive that their fathers' diet quality is worse than their own and their mothers' diet quality. Therefore, organizations that seek to provide adequate work conditions may not only positively influence their employees' JS and LS, but also their employees' adolescent children's LS. In parallel, public health campaigns promoting an adequate diet quality in workers, especially in male workers, may have a positive impact both on workers' health and wellbeing, and on their adolescent children' LS.

Lastly, the lack of crossover associations between parents' SWFaL and adolescents' LS may be due to the increased independence that adolescents seek from the family domain (51, 95). Adolescents seek sources of support outside of their family and may be less concerned with how their parents evaluate the family dynamics. In the context of the pandemic, it is possible that these relationships (albeit online) were more relevant than communication with their likely burdened parents, and therefore adolescents were unaware of their parents' perceptions about family issues.

Taken together, these crossover results suggest that mothers' LS is more influenced by their family members' domain satisfaction, particularly by their SWFaL. Furthermore, father-adolescent dyads had distinct crossover effects regarding JS and SWFoL. These findings suggest that the greater the mothers' involvement in family affairs and in traditional gender demands (23, 31, 61, 62), the more likely they may be to be influenced by both their male partner's and their adolescent children's satisfaction. Therefore, policy makers can develop organizational strategies to promote egalitarian gender roles among dual-earner parents, to give both members of these couples sufficient time to share and fulfill their family's responsibilities, especially during a public health crisis.

### 4.3. Gender differences in spillover and crossover effects

Although most spillover associations were stronger than crossover associations in the relationship between the three domain satisfactions and LS, there were gender differences in the job domain among parents. Consistent with expectations, the spillover between fathers' JS and LS was significantly higher than the crossover between mothers' JS and fathers' LS (H7a). Likewise, the spillover between mothers' JS and LS did not differ from the crossover between fathers' JS and mothers' LS (H7b). Although for mothers the crossover association between fathers' JS and mothers' LS was non-significant, mothers' LS seemed equally susceptible to their own JS as well as to fathers', whereas fathers' LS was more susceptible to their own JS than to mothers' JS. These results support pre-pandemic findings (25, 36) stressing the relevance of the work role for both the man's identity (65) and for their female partner in Latin American countries (36). Furthermore, spillover associations between each parent's SWFaL and LS were significantly higher than the crossover associations (H8a and H8b). That is, mothers' and fathers' LS were more susceptible to their own than to their partner's SWFaL, supporting the high relevance of family relationships in Latin-American countries (36, 66). In the food domain, in which mother-father crossover associations were non- significant, comparisons by gender indicate that for both parents LS is more susceptible to their own rather than to their partner's SWFoL (H9a and H9b). We hypothesize that a greater involvement of fathers on food-related tasks during the pandemic may increase the salience of the food domain for them, which in turn may increase the contribution that their SWFoL makes to their own LS.

Contrary to the expectations regarding the father's traditional role as the main provider for the family (27, 59), comparisons by gender indicate that adolescents' LS was equally influenced by both parents' JS (H10). Similar results were found for crossover from both parents' SWFaL and SWFoL to adolescents' LS (H11a and H11b). These latter results may be related to pandemic-related conditions. The restrictions on mobility imposed during the pandemic may have increased parent-adolescent interaction (see number of family meals), fathers' involvement in food and family issues (18, 20, 28), or blurred parents' gendered roles; thus adolescents may perceive an equal influence from both their mother and father. However, further research is needed to corroborate if these results are a consequence of the pandemic or represent a change in parents' gender roles.

### 4.4. The moderating roles of parents' job-related variables and city of residence

Our research question addressed the potential moderation of job-related variables in the above spillover and crossover relations. Examining mothers' type of employment as a moderator, results show that the association between mothers' SWFaL and fathers' LS, and between mothers' and fathers' SWFoL and fathers' LS was significant for self-employed mothers, and non-significant for employed mothers. Likewise, the association between fathers' JS and mothers' LS, and mothers' SWFoL and fathers' LS, was significant for self-employed fathers, and non-significant for employed fathers. In this sense, self-employed individuals have reported higher levels of LS (67) and JS (68, 69), which are associated with autonomy at work, flexibility, personal responsibility in task completion, safe working conditions and friendly atmosphere at work (68). Evidence also indicates that self-employed mothers experience greater SWFaL than employed mothers (70); while self-employed fathers experience greater work-life balance, which has been associated with a higher involvement in food-related tasks that increase both SWFoL and LS in their female partners (25, 61). Overall, self-employed parents may be more able to reconcile work and family life and related tasks such as food-related chores (68). Our results thus suggest that positive work conditions offered by self-employment not only enhance one parent's domain and overall life satisfaction, but also that of their partner.

Lastly, mothers' working hours moderated the association between fathers' JS and mothers' LS, and the association between fathers' JS and adolescents' LS. This association was non-significant when mothers had full-time jobs, and it was stronger when mothers worked part-time (<45 h/week). Mothers with part-time jobs are likely to have a lower monthly income than those with full-time jobs, and thus fathers' job and income might be of greater importance to the family (24, 36). This dynamic may help explain a stronger the associations between fathers' JS and their own and the adolescents' LS.

The family's city of residence moderated the association between mothers' family life satisfaction and father's life satisfaction, namely, this association remained non-significant in Rancagua whereas it became significant in Temuco, which aligned with results reported in a pre-pandemic study in this latter city (25). Previous studies during the pandemic have reported different levels of life satisfaction associated with city size, living in rural or urban areas and the region of residence, mainly due to conditions experienced during this health crisis, such as the proportion of people who suffered COVID-19, population size, more or less mobility and the house size (13, 73). In this regard, Temuco and Rancagua had similar cumulative incidence rates of COVID-19, amount of population and house sizes (96), and both cities were in mandatory lockdown for 2 months during the data collection period. Therefore, the pandemic conditions may not be sufficient to explain the different strength of the relationship between mothers' satisfaction with family life and fathers' life satisfaction, nor the significantly higher life satisfaction in fathers from Temuco compared with fathers from Rancagua. It is possible to hypothesize that the higher life satisfaction in fathers from Temuco may not only be positively influenced by their own family life satisfaction, but also by that of their female partners'. The city of residence also moderated the association between fathers' job satisfaction and the adolescent's life satisfaction, making this relationship non-significant for fathers living in Rancagua and stronger for those in Temuco. The difference in the strength of this association may relate to the finding that fathers from Temuco scored significantly higher in job satisfaction than fathers from Rancagua. Therefore, lower job satisfaction in fathers from Rancagua may not be enough to positively influence the adolescents' life satisfaction, while the opposite trend may occur in fathers living in Temuco. Nevertheless, further research is needed to explain these differences by city.

Overall, these results suggest that some dual-earner families are more vulnerable than others, such as families with employed parents and those living in Rancagua. Therefore, strategies to enhance dual-earner parents families' life domains or overall life satisfaction should have a special focus on families with employed parents, while differentiated strategies should be developed according to the main problems associated to the city of residence.

This study is not without limitations. First, this study had a cross-sectional design that does not allow to establish causality, and the sample was non-probabilistic. Future research should expand to longitudinal designs and include representative samples regarding family composition [i.e., socioeconomic status, family size, number of children, see (97, 98)]. In this regard, the samples were selected from a population of adolescents attending school, and thus another limitation is that these samples did not include families whose children were not in school. Another limitation is that some of the findings regarding gender dynamics may be specific to the Chilean context, and thus cross-cultural studies are needed to test these relationships in other Latin American countries and in others with diverging levels of gender equality. This research was designed and initiated before the COVID-19 pandemic, and thus specific conditions related to this context, such as working from home, were not explored. In addition, parents were only asked about the number of hours worked per week, but they were not asked if their job was based on shifts nor the hours of the shifts. Furthermore, although the moderating role of the city of residence was assessed, data was collected on different periods in each city which may have affected the results due to the different conditions of the pandemic in each city during 2020. Lastly, this study analyzed spillover and crossover associations between three domains and overall life satisfaction, whereas the moderating role of sociodemographic characteristics of the families, such as the parents' age, adolescents' gender, the number of children or income, was not assessed. Further studies must include other life domains such as health, leisure, friends, among others, and must evaluate the moderating role of work shifts–considering morning or afternoon shift, morning and afternoon or night shift- and the moderating roles of sociodemographic characteristics of the families.

### 4.5. Conclusions

This study provides new insights about the contribution of domain satisfaction to life satisfaction by analyzing spillover and crossover associations in dual-earner parents with adolescents. Our results show that each parent's life satisfaction may not only be influenced by their own satisfaction in a life domain, but it may also be influenced by the other parent's and their adolescent's domain satisfaction. In addition, while all spillover associations were positive, there were positive and negative crossovers between domain satisfaction and LS between parents and adolescents. These findings also contribute to the knowledge about gendered patterns in the relationship between domain satisfaction and life satisfaction in dual-earner parents with adolescents, as there were diverging spillover and crossover associations between mothers and fathers in the job domain. Lastly, our results show that job-related variables of both parents (parents' type of employment and mothers' working hours) and the city of residence moderated some spillover and crossover associations between each parent's JS, SWFaL and SWFoL on their own life satisfaction, and on that of the other parent and their adolescent children.

These results have research implications. As our results show that parents' job-related variables moderated some spillover and crossover associations between each parent's JS, SWFaL and SWFoL on their own, their partner's, and their adolescents' LS, future studies should also explore the moderating role of these variables on spillover and crossover associations between adolescents' SWFaL and SWFoL on their own and their parents' LS. These studies should also include other life domains such as health, peers, leisure, and school. In addition, future research should assess the moderating role of other parental job-related variables such as support from supervisors and coworkers, family-friendly organizational policies, autonomy, and flexibility at work.

## Data availability statement

The raw data supporting the conclusions of this article will be made available by the authors, without undue reservation.

## Ethics statement

The studies involving human participants were reviewed and approved by Ethics Committee of Universidad de La Frontera (protocol 007/19). Written informed consent to participate in this study was provided by the participants' legal guardian/next of kin.

## Author contributions

BS conceived of and wrote the first manuscript draft and approved the statistical analysis and the final version of the manuscript. AC-S and EM-Z guided the statistical analysis. LO, MS, KB, LR, and HP supervised data collection. LO, GL, ML, and CA-B made a critical analysis of the final version of the manuscript. All authors read and approved the final manuscript.
